# An association study of the *COL1A1* gene and high myopia in a Han Chinese population

**Published:** 2011-12-24

**Authors:** Dingding Zhang, Yi Shi, Bo Gong, Fei He, Fang Lu, He Lin, Zhengzheng Wu, Jing Cheng, Bin Chen, Shihuang Liao, Shi Ma, Jianbin Hu, Zhenglin Yang

**Affiliations:** 1 Sichuan Key Laboratory for Disease Gene Study, Sichuan Academy of Medical Sciences & Sichuan Provincial People’s Hospital, Sichuan, China; 2 Department of Laboratory Medicine, Sichuan Academy of Medical Sciences & Sichuan Provincial People’s Hospital, Sichuan, China; 3 Department of Ophthalmology, Sichuan Academy of Medical Sciences & Sichuan Provincial People’s Hospital, Sichuan, China

## Abstract

**Purpose:**

Single nucleotide polymorphisms (SNPs) in the collagen type I (*COL1A1*) gene have been shown to be significantly associated with high myopia in a Japanese population. This present study was conducted to investigate whether *COL1A1* is associated with high myopia in a Han Chinese population.

**Methods:**

High myopia is defined by a spherical equivalent of less than or equal to −6.00 diopter sphere and an axial length longer than or equal to 26.0 mm in the affected eye. We genotyped rs2075555 and rs2269336 SNPs in *COL1A1* in a Ha n Chinese group composed of 697 high myopia patients and 762 normal controls.

**Results:**

Neither of the two SNPs showed significant association with high myopia (p_allelic_=0.252 for rs2075555, and p_allelic_=0.699 for rs2269336).

**Conclusions:**

Our study revealed that SNPs in *COL1A1* are not significantly associated with high myopia in the Han Chinese population.

## Introduction

Myopia is the most common ocular disorder in the world. It causes light rays to focus on the front of the retina, and close objects are seen more clearly than distant objects. The prevalence of myopia is about 20%–30% in North American, Australian, and European populations [1-3], and much higher (40%–70%) in the Asian population [4-6], especially in China [7-9]. High myopia is an extreme form of myopia and one of the significant causes of blindness. It is characterized by a spherical equivalent of less than or equal to −6.00 diopter sphere and an axial length longer than or equal to 26.0 mm in the affected eye. The prevalence of high myopia is 1%–2% in the general population. High myopia has long been known to pose a high risk for the development of sight-threatening eye diseases, including glaucoma, macular hemorrhage, choroidal neovascularization, and retinal detachment [10].

The pathogenesis of myopia remains unclear. It has been reported that high myopia is a common eye disorder caused by the interaction of multiple genetic and environmental risk factors. Environmental risk factors such as high population density, near work, educational level, or economic development have been thought to explain higher prevalence of myopia among groups of people [11,12]. However, environmental risk factors cannot explain all the cases. Generally speaking, parents with myopia are more likely to have a child with myopia. Genetic factors responsible for high myopia are supported by many studies [13-19]. Twenty-one loci responsible for myopia have been mapped by whole genome linkage analysis and genome-wide association study [20-24].

The collagen type I (*COL1A1*) gene has been described as playing an important role in the pathogenesis of experimental myopia [25-28]. *COL1A1* is located on chromosome 17q21 where the myopia 5 (MYP5) locus was identified [29]. Type I collagen is encoded by this gene. Previous studies suggested that dysfunction of type I collagen genes was associated with disorders such as osteogenesis imperfecta, systemic diseases with scleral thinning, and myopia [30-32]. Single nucleotide polymorphisms (SNPs) in *COL1A1* showed highly significant genotypic association with high myopia in a Japanese population [33]. However, this finding could not be replicated by other studies in Chinese, Caucasian, even in the Japanese population [34-36]. To further determine the association between *COL1A1* and high myopia, we studied the association of *COL1A1* with high myopia in a Han Chinese population composed of 697 subjects with high myopia and 762 matched normal controls.

## Methods

### Subjects

This study was approved by the Institutional Review Boards of the Sichuan Academy of Medical Sciences & Sichuan Provincial People’s Hospital, Sichuan, China. All subjects provided informed consent before participating in the study. Subjects with syndromic disorders or systemic diseases that could lead to myopia were excluded from among the participants. All participants went through a standard ophthalmic examination protocol. High myopia is defined by a spherical equivalent of less than or equal to −6.00 diopter sphere (DS) and an axial length longer than or equal to 26.0 mm in affected patients’ eyes. In some cases, high myopia may also include degenerative changes at the back of the eye, such as retinal damage or detachment. In total, 697 high myopia patients (Spherical refraction ≤-6.00D), including 276 patients with high myopia of −9.25D or less, and 762 matched normal controls were recruited from Sichuan Academy of Medical Sciences & Sichuan Provincial People’s Hospital. In the matched normal controls, all participants underwent an eye exam and were found to have no signs of early myopia (spherical equivalent >-1.0 DS). Clinical information about the cases and controls is listed in Table 1.

**Table 1:** Characteristics of high myopia cases and controls in this study.

**Groups**	**Number**	Age (Years)*	**Gender**		**Refractive errors (Diopter, ±)**	**Axial length (mm)**
** **	** **	** **	**Male**	**Female**	** **	** **
Cases	697	34.3 ±11.9	317	380	−9.77 ±3.35 (OD), −9.82 ±3.21 (OS)	27.64 ±1.75 (OD), 27.83 ±1.79 (OS)
Controls	762	54.5 ±9.2	351	411	** **	** **

*The age when the cases and controls were recruited. ±: standard deviation; OD: right eye; OS: left eye; mm: millimeter.

### SNP selection and genotyping

We selected rs2075555 and rs2269336 at the *COL1A1* locus to genotype in the Han Chinese population as these two SNPs were significantly associated with high myopia in a Japanese population [33]. Venous blood from each subject was drawn and collected in an EDTA tube. Genomic DNA was extracted from the blood by serial phenol/chloroform extraction and ethanol precipitation. SNP genotyping was performed with the dye terminator-based SNaPshot method (Applied Biosystems, Foster City, CA). SNP analysis was performed on the ABI 3130 Genetic Analyzer (Applied Biosystems). The SNP reported in this manuscript has a genotyping success rate of 97% accuracy as judged by random re-genotyping of 10% of the samples in the cohort. For rs2075555, the PCR forward primer 5′-GCC CTT CCT TGT CTT CTT-3′, PCR reverse primer 5′-ACC GCC ATC CCT TTG TTT-3′, and SNaPshot primer 5′- GCC TCT TCC CCA AAA GAT −3′ were used in genotyping. For rs2269336, the PCR forward primer 5′-TCC CCT TTG CCT TCG TTG-3′, PCR reverse primer 5′-AAG CCC CTT CTC CAG TTG-3′, and SNaPshot primer 5′-GAA TGG GAC ATG GAG GAA GAA AGG ACG TGG AGT TCT AGA G-3′, were used in genotyping. In brief, the polymerase chain reactions (10 μl ﬁnal volume) contained 50 ng of genomic DNA, 1μl of each primer (10 pmol/μl), 1 μl of 10 buffer (Takara Bio Inc., Shiga, Japan), 0.8 μl of deoxyribonucleotide triphosphates (2 mmol/l; Takara Bio Inc.), 0.4 μl MgCl_2_ (2.5 mmol/l; Takara Bio Inc.), and 0.1 μl of ExTaq polymerase (5 U/μl; Takara Bio Inc.). The product was then processed per the ABI SNaPshot protocol using primers designed for ﬂuorescent dideoxy nucleotide termination.

### Statistical analysis

The Hardy–Weinberg equilibrium (HWE) for each SNP polymorphism was tested by the χ^2^ test. All analyses were adjusted for matching factors of age and gender. P values of the SNPs were calculated using an additive model. The unadjusted odds ratios of alleles and genotypes between cases and controls were estimated by the χ^2^ test. All statistical analyses were performed using the software SPSS 10.0 (SPSS Inc., Chicago, IL).

## Results

The two selected SNPs were successfully genotyped and were within HWE in both case and control groups (p>0.05). The SNP frequencies in this study were similar to those of Han Chinese Beijing (HCB) available in HapMap3, which implied reliable genotyping data in the study. Neither of the two SNPs showed significant association between high myopia (Spherical refraction ≤-6.00D) and controls (p>0.05), the association results of the SNPs (rs2075555 and rs2269336) in *COL1A1* and high myopia in a Han Chinese cohort are listed in Table 2 and Table 3, respectively. Based on the odds ratio in previous study in the Japanese [33], the power of a hypothesis test for two SNPs (rs2075555 and rs2269336) were 83% and 77% in this study using SAS 9.2 (SAS/Genetics; SAS Institute, Cary, NC), suggesting a sufficient power to reject the null hypothesis of no association between the two SNPs in *COLA1* and high myopia. Furthermore, the two SNPs showed no significant association (p>0.05) with high myopia of −9.25D or less as the definition of high myopia in the previous study [33] (Table 4 and Table 5).

**Table 2:** Association between rs2075555 in *COL1A1* and high myopia (≤-6.00 D) in a Han Chinese population.

**Phenotype**	**HWE**	**Genotype count(frequency)**	**Allele frequency**	**Allelic p**	**OR (95%CI)**	**Trend p value**
Case (697)	0.79	AA: 91 (0.13)	A: 0.37	0.252	1.09 (0.94–1.30)	0.239
** **	** **	AC: 333 (0.48)	C: 0.63	** **	** **	** **
** **	** **	CC: 273 (0.39)	** **	** **	** **	** **
Control (762)	0.09	AA: 79 (0.10)	A:0.35	** **	** **	** **
** **	** **	AC: 374 (0.49)	C:0.65	** **	** **	** **
** **	** **	CC: 309 (0.41)	** **	** **	** **	** **

**Table 3:** Association between rs2269336 in *COL1A1* and high myopia (≤-6.00 D) in a Han Chinese population.

**Phenotype**	**HWE**	**Genotype count(frequency)**	**Allele frequency**	**Allelic p**	**OR (95%CI)**	**Trend p value**
Case (697)	0.22	CC: 254 (0.36)	C:0.59	0.699	1.03 (0.80–1.19)	0.697
** **	** **	CG: 315 (0.45)	G:0.41	** **	** **	** **
** **	** **	GG: 128 (0.18)	** **	** **	** **	** **
Control (762)	0.05	CC: 243 (0.32)	C:0.58	** **	** **	** **
** **	** **	CG: 403 (0.53)	G:0.42	** **	** **	** **
** **	** **	GG: 116 (0.15)	** **	** **	** **	** **

**Table 4:** Association between rs2075555 in *COL1A1* and high myopia (≤-9.25 D) in a Han Chinese population.

**Phenotype**	**HWE**	**Genotype count(frequency)**	**Allele frequency**	**Allelic p**	**OR (95%CI)**	**Trend p value**
Case (276)	0.91	AA: 38 (0.14)	A: 0.37	0.311	1.11 (0.90–1.36)	0.296
** **	** **	AC: 130 (0.47)	C: 0.63	** **	** **	** **
** **	** **	CC: 108 (0.39)	** **	** **	** **	** **
Control (762)	0.09	AA: 79 (0.10)	A:0.35	** **	** **	** **
** **	** **	AC: 374 (0.49)	C:0.65	** **	** **	** **
** **	** **	CC: 309 (0.41)	** **	** **	** **	** **

**Table 5:** Association between rs2269336 in *COL1A1* and high myopia (≤-9.25 D) in a Han Chinese population.

**Phenotype**	**HWE**	**Genotype count(frequency)**	**Allele frequency**	**Allelic p**	**OR (95%CI)**	**Trend p value**
Case (697)	0.30	CC: 107 (0.39)	C:0.61	0.266	1.12 (0.92–1.37)	0.254
** **	** **	CG: 123 (0.45)	G:0.39	** **	** **	** **
** **	** **	GG: 46 (0.17)	** **	** **	** **	** **
Control (762)	0.05	CC: 243 (0.32)	C:0.58	** **	** **	** **
** **	** **	CG: 403 (0.53)	G:0.42	** **	** **	** **
** **	** **	GG: 116 (0.15)	** **	** **	** **	** **

## Discussion

In high myopia, there is the risk of sight loss because the deformation of the eye provokes stress on the retina, which can become detached and can also lead to other changes, including macular hemorrhage, glaucoma, and retinal atrophy degeneration. The condition demands study because of its severe clinical consequences and its high prevalence in the world. Previous studies indicated that high myopia is associated with marked scleral thinning and ocular axial elongating [37]. Changes in scleral collagen appear to be involved in the development of myopia [38,39].

However, there is still some controversy regarding the association of *COL1A1* and high myopia. One case-controlled association study genotyped ten SNPs in a Japanese population of 330 subjects with high myopia of −9.25D or less and 330 randomized controls without high myopia [33]. The study found two SNPs (rs2075555 and rs2269336) were significantly associated with high myopia (p<0.05, p<0.01) and showed that *COL1A1* was associated with high myopia. Additionally, a separate case-controlled study composed of 471 high myopia cases and 623 controls, demonstrated that there is no significant association with the polymorphisms of *COL1A1* and high myopia in the Taiwanese population [35]. A third study, which comprised 427 high myopia cases and 420 controls, analyzed eight tag SNPs, including rs2075555 and rs2269336, to tag the linkage disequilibrium blocks harboring *COL1A1* [34]. The study identified an absence of association between *COL1A1* polymorphisms and high myopia in the Japanese population. Finally, a study of the association between SNPs in the *COL1A1/COL2A1* gene and high myopia in Caucasian family data sets comprising 146 (Duke) and 130 (Cardiff) families with high myopia found that *COL1A1* gene variants were not associated with myopia, while *COL2A1* was associated with high myopia in two independent Caucasian family data sets [36].

In this study, we investigated the association between SNPs in *COL1A1* and high myopia. Generally, the definition of high myopia is a spherical equivalent of less than or equal to −6.00 diopter sphere and an axial length longer than or equal to 26.0 mm in the affected eye. We genotyped rs2075555 and rs2269336 SNPs in *COL1A1* in a Han Chinese group composed of 697 high myopia patients (spherical equivalent:-6.00 OD or less) and 762 controls. Neither of the two SNPs showed significant association with high myopia (p>0.05). Consistent with the results in Caucasian and Taiwanese populations, our results failed to identify *COL1A1* as a significant risk factor for high myopia in the mainland Han Chinese population. The definition of high myopia in the original study by Inamori et al. [33] was a spherical equivalent of less than or equal to −9.25 diopter sphere. We further analyzed the association between the two SNPs and patients with −9.25 or less diopter sphere (276 cases) and 762 controls, we did not see any significant associations either. This suggests that possible heterogeneity among different ethnicities or genetic variants in the *COL1A1* gene have nothing to do with high myopia.
